# Distal nerve transfers for radial nerve reinnervation and hand function restoration

**DOI:** 10.1016/j.bas.2026.106026

**Published:** 2026-03-28

**Authors:** Andrija Savić, Jovan Grujić, Aleksandar Djurdjević, Svetozar Stanković, Aleksa Mićić, Aleksandra Stojiljković, Branko Gaković, Milan Lepić, Nenad Novaković, Lukas Rasulić

**Affiliations:** a Faculty of Medicine, University of Belgrade, Belgrade, Serbia; b Clinic for Neurosurgery, University Clinical Centre of Serbia, Belgrade, Serbia; c Medical Faculty, Military Medical Academy, University of Defence, Belgrade, Serbia; d Clinic for Neurosurgery, Military Medical Academy, Belgrade, Serbia; e Clinic for Neurosurgery, University Clinical Centre of Kragujevac, Kragujevac, Serbia; f Clinic for Vascular Surgery, University Clinical Centre of Serbia, Belgrade, Serbia

**Keywords:** Radial nerve injury, Distal nerve transfer

## Abstract

**Introduction:**

Radial nerve palsy, with wrist drop and loss of finger and thumb extension, severely impairs grasp and release. In proximal or extensive defects, direct repair or grafting may fail, making distal nerve transfer a preferred alternative.

**Research question:**

Can distal median-to-radial nerve transfers restore hand extension and improve quality of life in patients with radial nerve injury?

**Material and methods:**

This prospective study included nine patients with radial nerve injuries treated using distal nerve transfer. Surgical reconstruction consisted of transfer of the median nerve branch to the flexor digitorum superficialis to the radial nerve branch for the extensor carpi radialis brevis, combined with transfer of the median nerve branch to the flexor carpi radialis to the posterior interosseous nerve. In addition, a pronator teres to extensor carpi radialis brevis tendon transfer was performed to facilitate wrist extension in long-standing lesions. Functional recovery of wrist, finger (II–V), and thumb extension was assessed 24 months postoperatively using a modified British Medical Research Council scale, and quality of life was evaluated preoperatively and at final follow-up using the PNSQoL questionnaire.

**Results:**

All patients achieved satisfactory recovery (BMRC M3–M5) of wrist, finger, and thumb extension, with M4–M5 outcomes predominating, especially for wrist extension. Median PNSQoL improved from 49 to 78, with 88.9% reporting excellent quality of life.

**Discussion and conclusion:**

Distal median-to-radial nerve transfer reliably restores hand extension and improves patient-reported outcomes in proximal radial nerve injuries, though larger multicenter studies are needed for confirmation.

## Highlights

• Distal nerve transfer is effective for proximal radial nerve injuries.
• Median-to-radial transfers restore wrist, finger, and thumb extension.
• All patients achieved satisfactory motor recovery at 24 months.
• Patient-reported quality of life improved significantly after surgery.

## 1 Introduction

The clinical picture of the radial nerve injury is characterized by a wrist drop deformity and this significantly impairs the quality of life of these patients. Wrist extension is a necessary prerequisite for effectively grasping objects and on the other hand, finger extension is directly responsible for releasing objects. As a result, patients with radial nerve injuries have difficulty performing basic life activities such as personal hygiene, dressing, and taking water and food. (Rasulic et al., 2017).

In majority of cases, radial nerve injuries can be treated with one of the methods of direct nerve repair. But in a certain number of cases, the defect in the nerve substance is extremely large, so that reconstruction of the function of the injured nerve is possible only by applying the method of distal nerve or tendon transfer (Rasulić et al., 2017).

Distal nerve transfers imply the use of the terminal lateral branches of the nerves as the donors, whereby the sacrifice of the donor branch does not lead to the appearance of a functional deficit. On the other side recipients are the lateral terminal branches of the injured nerves which are very close to the target muscle which we want to reinnervate (Rasulić et al., 2018). In selected cases, pronator teres to extensor carpi radialis brevis tendon transfer may be used as an adjunct for early restoration of wrist extension, thus improving distal nerve transfers outcomes.

## 2 Method

### 2.1 Study design and patients

This prospective study included 9 patients (Appendix A) who were operated at the Clinic for Neurosurgery, Clinical Centre of Serbia, between 1st January 2010 and 31st December 2016 using distal nerve transfer for radial nerve reconstruction.

The distal nerve transfer procedures included:

• The transfer of the branch of the median nerve for the flexor digitorum superficialis muscle to the branch of the radial nerve for the extensor carpi radialis brevis muscle.
• The transfer of the branch of the median nerve for the flexor carpi radialis muscle to the posterior interosseus nerve.

In selected cases (3), surgical reconstruction also included a pronator teres to extensor carpi radialis brevis tendon transfer to augment wrist extension. This transfer was employed as an adjunct to nerve transfers in long-standing lesions, including both isolated radial nerve injuries and infraclavicular brachial plexus lesions with predominant radial nerve involvement.

Three weeks after the surgery, all the patients underwent physical therapy in the duration of at least 12 weeks. The final evaluation of the operative treatment was done two years after the surgery.

This study excluded patients who, in addition to peripheral nerve injuries of the upper extremities, also had peripheral nerve injuries of the lower extremities or cranial nerve injuries. Patients were also excluded if they did not provide written consent to participate, failed to undergo at least 12 weeks of postoperative physical therapy, or did not attend follow-up examinations for a minimum of two years after surgery.

In the postoperative evaluation of hand function, the following was analyzed: wrist extension, 2nd to 5th finger extension, thumb extension, and the patients’ quality of life.

A slightly modified version of the British Medical Research Council Scale (BMRC) was used for the assessment of the strength of wrist extension, 2nd to 5th finger extension, and thumb extension. Additionally, M0, M1, and M2 were considered unsatisfactory, while M3, M4, and M5 were designated as satisfactory useful functional recovery. In the group of functionally satisfactory recoveries, M5 is marked as excellent, M4 as very good, and M3 as good.

### 2.2 Quality of life (QOL)

QOL was assessed using the Peripheral Nerve Surgery Quality of Life (PNSQOL) questionnaire. Scores range from 0 to 80 and are categorized as poor (0–39), fair (40–49), good (50–59), very good (60–69), and excellent (70–80) (***Apendix B***). The questionnaire was administered preoperatively and 24 months postoperatively, and results were compared.

### 2.3 Surgical procedure

**Fig. 1:**
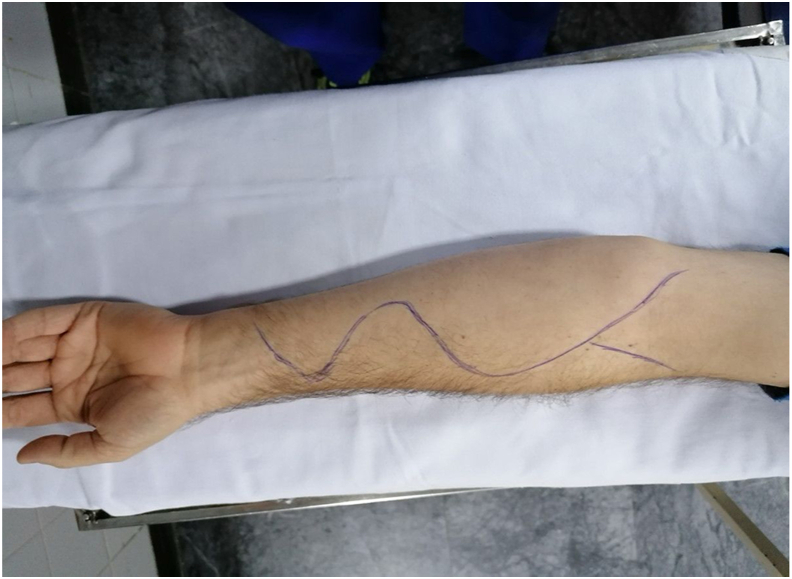
Skin incision at the level of the forearm, marked along the projected course of the donor and recipient nerves for distal nerve transfer.

With the patient supine and the arm in abduction, an S-shaped incision is made along the anterior forearm, extending from the proximal to the middle third. A proximal linear extension of the incision along the brachioradialis provides access to the lateral bicipital groove (Fig. 1).

**Fig. 2:**
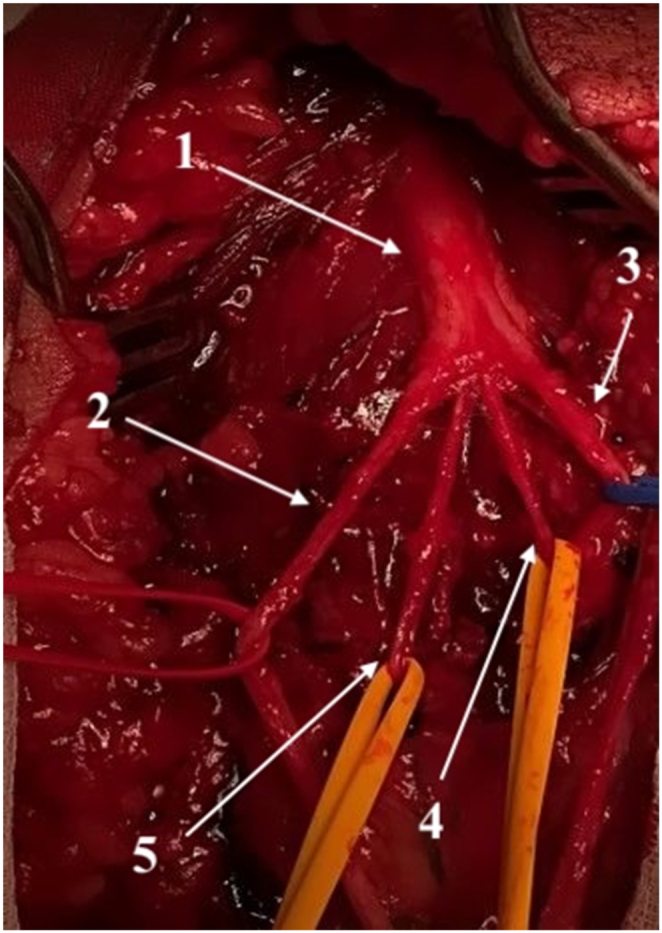
Nervus radialis and its branches at the level of the elbow. (1) Radial nerve, (2) superficial branch of the radial nerve, (3) posterior interosseous nerve, (4) branch to the supinator muscle, and (5) branch to the extensor carpi radialis brevis muscle.

**Fig. 3:**
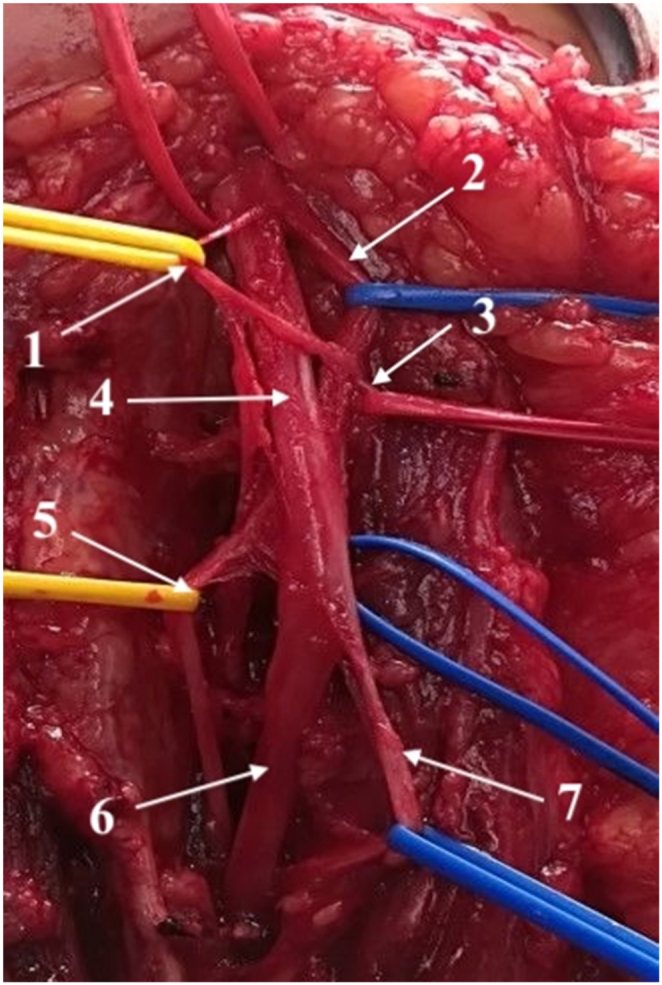
Median nerve and its branches at the level of the elbow and proximal forearm. (1) Branch to the pronator teres muscle, (2) branch to the flexor carpi radialis muscle, (3) proximal branch to the flexor digitorum superficialis muscle, (4) median nerve, (5) anterior interosseous nerve, (6) remaining median nerve containing sensory fibers for protective sensibility and motor fibers to the thenar muscles, and (7) distal branch to the flexor digitorum superficialis muscle.

**Fig. 4:**
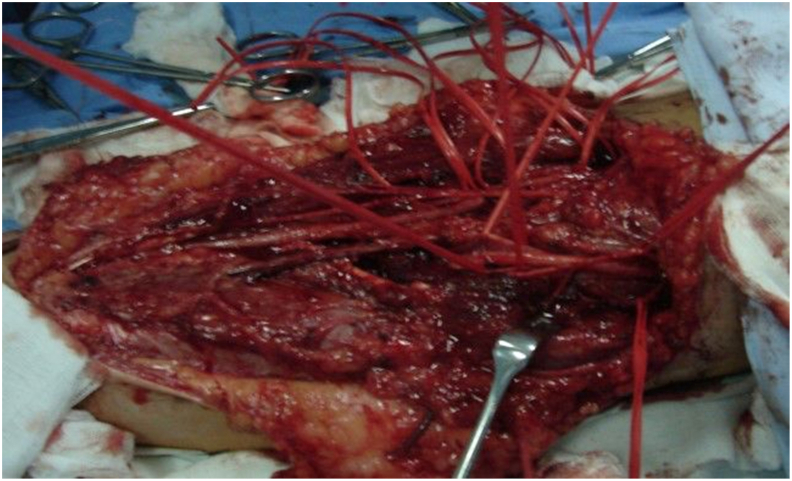
Radial and median nerves with their terminal branches.

**Fig. 5:**
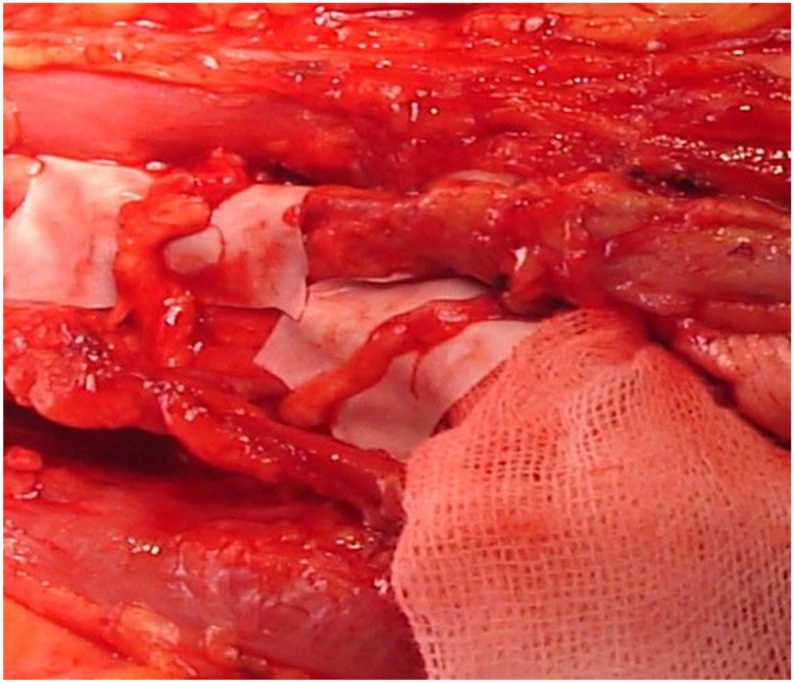
Direct nerve coaptation.

Skin and subcutaneous tissue are divided, preserving the medial and lateral cutaneous nerves of the forearm and the cephalic vein. The biceps aponeurosis is resected and the intermuscular fascia opened. Retracting the brachioradialis laterally exposes the superficial radial nerve (laterally) and the radial artery (medially). Deeper dissection reveals the pronator teres tendon, which is released with its periosteal insertion for later transfer to the extensor carpi radialis brevis (ECRB) (Fig. 2). This step relaxes the pronator teres, enabling medial retraction and access to the proximal median nerve branches. The pronator teres muscle is mobilized from the radial artery, with transverse arterial branches cauterized. The median nerve trunk is identified and traced distally. Branches to the flexor carpi radialis (FCR, often including palmaris longus), pronator teres, and flexor digitorum superficialis (FDS) are dissected. Of these, the FCR branch is designated for transfer to the posterior interosseous nerve (PIN), and a proximal FDS branch is used for transfer to the ECRB branch. Donor branches are prepared as distally as possible to maximize length (Fig. 3). The superficial radial nerve, PIN, and ECRB branch are dissected proximally to the radial nerve bifurcation and released distally to ensure mobility. The ECRB branch is thinner and nearly parallel to the superficial radial nerve, while the PIN is thicker and courses deeper and more medially (Fig. 4). The FCR branch is transected distally and coapted to the PIN. The proximal FDS branch is similarly coapted to the ECRB branch. Recipients are divided proximally to allow for tension-free anastomosis (Fig. 5). As an adjunct, the pronator teres tendon is transferred to the ECRB tendon to augment wrist extension.

## 3 Results

The study included nine patients who underwent distal nerve transfer for radial nerve reconstruction. The cohort consisted of seven men (77.8%) and two women (22.2%), with a median age of 35 years (interquartile range [IQR] 26–39, range 18–48). The median time from injury to surgery was 6 months (IQR 4–7). The mechanisms of injury included traffic accidents (3 cases), fall from height (1 case), penetrating injury (1 case), and iatrogenic injury (4 cases). All patients completed a minimum of 12 weeks of postoperative physical therapy and were available for clinical and functional evaluation at a minimum follow-up of 24 months.

Quality of life, assessed using the PNSQOL questionnaire, showed a marked improvement following surgery. Preoperatively, most patients reported poor to good quality of life, with no patients achieving very good or excellent scores. At 24-month follow-up, a substantial shift toward higher PNSQOL categories was observed, with the majority of patients reporting excellent quality of life. Median PNSQOL score improved from 49 (IQR 45–54) preoperatively to 78 (IQR 76–80) at final follow-up, which was statistically significant (Table 1).

**Table 1:** Distribution of PNSQOL categories preoperatively and at 24-month follow-up, demonstrating a shift toward excellent quality-of-life outcomes following distal nerve transfer.

PNSQOL category	Score range	Preoperative n (%)	24-month follow-up n (%)
Poor	0–40	1 (11.1%)	0 (0%)
Fair	40–50	4 (44.4%)	0 (0%)
Good	50–60	3 (33.3%)	0 (0%)
Very good	60–70	1 (11.1%)	1 (11.1%)
Excellent	70–80	0 (0%)	8 (88.9%)

Functional recovery was assessed at 24 months postoperatively using a modified British Medical Research Council (BMRC) scale. All patients achieved satisfactory functional recovery (BMRC M3–M5) for wrist extension, finger extension (II–V), and thumb extension. The majority of patients demonstrated very good to excellent outcomes (M4–M5), with the highest rates of excellent recovery observed in wrist extension. No patients remained in the unsatisfactory recovery group (M0–M2) for any assessed function at final follow-up (Table 2).

**Table 2:** Functional outcomes assessed at the 24-month follow-up demonstrated satisfactory recovery (BMRC M3–M5) in all patients for wrist extension, finger extension (II–V), and thumb extension. Very good outcomes (M4) predominated across all assessed functions, while excellent recovery (M5) was most frequently observed in wrist extension. No unsatisfactory functional outcomes (M0–M2) were identified at final evaluation.

Function assessed	M3 n (%)	M4 n (%)	M5 n (%)	Satisfactory recovery (M3–M5)
Wrist extension	1 (11.1)	5 (55.6)	3 (33.3)	9/9 (100%)
Finger extension II–V	2 (22.2)	5 (55.6)	2 (22.2)	9/9 (100%)
Thumb extension	3 (33.3)	5 (55.6)	1 (11.1)	9/9 (100%)

## 4 Discussion

There are studies indicating that excellent results can be achieved in radial nerve lesions through the application of grafting procedures (Samardzić et al., 1990; Bertelli and Ghizoni, 2016). Although in some cases favorable outcomes can be achieved, the author of this study argues that treating proximal and extended defects of the radial nerve using nerve grafts alone remains debatable. The main concern is the long distance regenerating axons must traverse to reach target muscles, which often delays reinnervation until degenerative changes occur in the motor end plates. In addition, a proportion of regenerating axons may be misdirected into the superficial sensory branch at the terminal division of the radial nerve, further reducing the chance of adequate motor recovery. Large radial nerve defects often require harvesting the sural nerve from both legs, significantly prolonging surgery and recovery. Moreover, in traction injuries with preserved continuity but functional loss, grafting frequently proves ineffective. By contrast, distal nerve transfer eliminates the need for grafts and avoids the additional suture line, thereby reducing axonal loss. This approach effectively converts a proximal lesion into a distal one, markedly shortening the distance to the motor end plates. As a result, even patients presenting late after injury may remain suitable candidates for nerve reconstruction.

Although tendon transfer for the radial nerve is among the most effective reconstructive procedures, providing relatively rapid clinical improvement, the author of this study also emphasizes its disadvantages. These include disturbances in muscle biomechanics due to altered muscle attachment, orientation, and tension; scarring at the muscle origin from extensive dissection; the requirement for a wide passive range of motion; prolonged immobilization; the risk of tendon rupture; and the formation of adhesions between the transferred tendons and surrounding tissues, which may ultimately result in suboptimal function. Also, some authors report a reduction in grip strength and radial deviation of the hand in operated patients, particularly in those who underwent transfer of the flexor carpi ulnaris to the extensor digitorum communis (Ropars et al., 2006). On the other hand, the definitive results of nerve transfer are achieved only after a considerably longer period. This approach, however, enables restoration of hand function even in patients with swelling or stiffness associated with a limited passive range of motion. Importantly, nerve transfer provides a higher quality of functional recovery, allowing for independent and synchronized extension of the fingers and hand—a feature crucial for certain activities such as playing the piano (Davidge et al., 2013).

Different authors have used various combinations of nerve transfers in an attempt to reconstruct radial nerve function:

In a study by Garcia-Lopes et al. (García-López et al., 2014) patients with radial nerve injuries underwent transfer of the branch to the pronator teres muscle to the branch of the radial nerve for the extensor carpi radialis longus, combined with transfer of the median nerve branch to the flexor carpi radialis to the posterior interosseous nerve. The authors of this study note that the radial nerve branch to the extensor carpi radialis longus is typically harvested at a high level, in the lateral bicipital groove and proximal to the terminal branches of the radial nerve, making direct coaptation with the donor branch from the median nerve to the pronator teres technically challenging. In addition, wrist extension mediated by the extensor carpi radialis longus results in radial deviation, since its distal insertion at the base of the second metacarpal causes outward movement in addition to extension. Also, usage of pronator teres nerve branch limits utility of this muscle for tendon transfer to the extensor carpi radialis brevis, although an accessory median nerve branch to the pronator teres is frequently present.

On the other side in some studies (Mackinnon et al., 2007; Ray and Mackinnon, 2011), patients with radial nerve injuries underwent transfer of the median nerve branch to the flexor digitorum superficialis to the radial nerve branch for the extensor carpi radialis brevis, along with transfer of the median nerve branch to the flexor carpi radialis to the posterior interosseous nerve. According to the author of this study, this combination of nerve transfers—the median nerve branch to the flexor digitorum superficialis to the radial nerve branch for the extensor carpi radialis brevis, and the median nerve branch to the flexor carpi radialis to the posterior interosseous nerve—represents the ideal configuration for distal nerve transfer in radial nerve injuries. The donor and recipient nerves exhibit synergistic action, facilitating significantly faster and easier postoperative re-education. Specifically, wrist extension increases the passive tension of the finger flexor tendons, resulting in their contraction, with wrist extension followed by finger flexion at the interphalangeal joints—a natural position for grasping. This explains the synergism between the flexor digitorum superficialis and the extensor digitorum brevis, or between the donor and recipient nerves that innervate them. Conversely, wrist flexion increases the passive tension of the finger extensor tendons, promoting finger extension, which accounts for the synergism between the flexor carpi radialis, extensor digitorum communis, and extensor pollicis longus, as well as the nerves that innervate them. Furthermore, the aforementioned donor and recipient nerves possess compatible histomorphology, allowing direct coaptation, optimal alignment of nerve trunk cross-sections, tension-free suturing, and a short distance for regenerating axons to reach the motor end plates of the target muscles (Caetano et al., 2018; Sukegawa et al., 2016; Ukrit et al., 2009). For the posterior interosseous nerve, resection of its branches to the supinator muscle is necessary, as this directs regenerating axons primarily toward the extensor digitorum communis and extensor pollicis longus, while preserving elbow supination through the biceps brachii. Additionally, decompression of the nerve at Frohse's arcade is required, as this site is a common location of secondary compression that can interfere with nerve regeneration (Johnston et al., 1993; Spinner, 1968). Performing these two procedures improves mobilization of the posterior interosseous nerve and facilitates a tension-free, direct coaptation.

Compared to finger extension, wrist extension requires greater force, and in many cases, the transfer of the median nerve branch to the flexor digitorum superficialis to the radial nerve branch for the extensor carpi radialis brevis must be reinforced with a tendon transfer of the pronator teres to the extensor carpi radialis brevis, particularly when a long interval exists between injury and surgery.

It is important to note that the flexor carpi ulnaris and flexor digitorum profundus provide sufficient strength for hand and finger flexion, so partial sacrifice of the flexor digitorum superficialis and complete sacrifice of the flexor carpi radialis does not result in functional deficits. In contrast, the extensor carpi radialis brevis has a thicker and stronger tendon than the extensor carpi radialis longus, with its insertion at the base of the third metacarpal—essentially at the midpoint of the wrist. This makes it the primary wrist extensor, allowing extension along the midline without radial or ulnar deviation.

Humeral fractures or osteosynthesis were frequent associated injuries due to the radial nerve's anatomical proximity to the humerus. Patients without associated injuries had more M5 recoveries, whereas those with additional injuries had more M3 recoveries.

In our series, patients with Sunderland grade V lesions (isolated radial nerve injury) achieved more M5 and M3 recoveries compared to grade IV lesions (infraclavicular brachial plexus lesions with predominant radial nerve involvement), while M4 recovery rates were similar. In grade V lesions, combining distal nerve transfer with nerve grafting increased M5 recoveries, and additional tendon transfer of pronator teres to extensor carpi radialis brevis enhanced M3 recoveries, especially in patients operated on later. Wrist extension outcomes often exceeded thumb and finger extension, with early recovery confirming tendon transfer efficacy.

All patients achieving restoration of wrist, thumb, and finger extension showed improved postoperative PNSQOL scores, indicating a significant positive impact on functional outcomes and quality of life.

The main limitation of this study is the small sample size and the absence of a blinded review; preoperative evaluation, surgical treatment, postoperative assessment, and data collection, processing, and analysis were all conducted by the study author. To draw more robust conclusions, larger multicenter studies with independent surgeons and researchers for data collection and interpretation are necessary. An additional limitation of the study is that early postoperative wrist extension was not systematically evaluated, which limits the ability to directly compare the early contribution of tendon transfer with the later recovery resulting from nerve regeneration.

## 5 Conclusion

Distal nerve transfers from median-to-radial nerve provided reliable restoration of hand extension in patients with proximal or extensive radial nerve injuries. In this series of nine patients, 100% achieved satisfactory motor recovery (≥M3) of wrist, finger, and thumb extension. Patient-reported quality of life improved markedly, with median PNSQoL scores increasing from 49 preoperatively to 78 postoperatively, and 88.9% of patients reporting excellent QoL at final follow-up. Despite these consistently favorable results, the current evidence base in peripheral nerve surgery remains largely limited to single-center series; therefore, meaningful advancement in evidence quality can only be achieved through multicentric collaboration and prospective study designs.

## Declaration of competing interest

The authors declare that they have no known competing financial interests or personal relationships that could have appeared to influence the work reported in this paper.
