# Supplementary material for: Distal nerve transfers for radial nerve reinnervation and hand function restoration
Source: Brain Spine. 2026 Mar 28;6:106026. doi: 10.1016/j.bas.2026.106026 (PMC13081166; doi:10.1016/j.bas.2026.106026)
Supplement: Multimedia component 1 [file mmc1.docx]

***Table 1****. Distribution of PNSQOL categories preoperatively and at 24-month follow-up, demonstrating a shift toward excellent quality-of-life outcomes following distal nerve transfer.*

| **PNSQOL category** | **Score range** | **Preoperative n (%)** | **24-month follow-up n (%)** |
| --- | --- | --- | --- |
| Poor | 0–40 | 1 (11.1%) | 0 (0%) |
| Fair | 40–50 | 4 (44.4%) | 0 (0%) |
| Good | 50–60 | 3 (33.3%) | 0 (0%) |
| Very good | 60–70 | 1 (11.1%) | 1 (11.1%) |
| Excellent | 70–80 | 0 (0%) | 8 (88.9%) |
