# Supplementary material for: Distal nerve transfers for radial nerve reinnervation and hand function restoration
Source: Brain Spine. 2026 Mar 28;6:106026. doi: 10.1016/j.bas.2026.106026 (PMC13081166; doi:10.1016/j.bas.2026.106026)
Supplement: Multimedia component 2 [file mmc2.docx]

***Table 2.*** *Functional outcomes assessed at the 24-month follow-up demonstrated satisfactory recovery (BMRC M3–M5) in all patients for wrist extension, finger extension (II–V), and thumb extension. Very good outcomes (M4) predominated across all assessed functions, while excellent recovery (M5) was most frequently observed in wrist extension. No unsatisfactory functional outcomes (M0–M2) were identified at final evaluation.*

| **Function assessed** | **M3 n (%)** | **M4 n (%)** | **M5 n (%)** | **Satisfactory recovery (M3–M5)** |
| --- | --- | --- | --- | --- |
| Wrist extension | 1 (11.1) | 5 (55.6) | 3 (33.3) | 9/9 (100%) |
| Finger extension II–V | 2 (22.2) | 5 (55.6) | 2 (22.2) | 9/9 (100%) |
| Thumb extension | 3 (33.3) | 5 (55.6) | 1 (11.1) | 9/9 (100%) |
